# Neuro‐Sjögren: Uncommon or underestimated problem?

**DOI:** 10.1002/brb3.1665

**Published:** 2020-06-25

**Authors:** Marta Jaskólska, Magdalena Chylińska, Anna Masiak, Mariusz Siemiński, Marcin Ziętkiewicz, Zenobia Czuszyńska, Żaneta Smoleńska, Zbigniew Zdrojewski

**Affiliations:** ^1^ Department of Internal Medicine Connective Tissue Diseases and Geriatrics Medical University of Gdansk Gdansk Poland; ^2^ Department of Adult Neurology Medical University of Gdansk Gdansk Poland

**Keywords:** antinuclear antibodies, autoimmune neuropathy, autoimmunity, Sjögren's syndrome, small‐fiber neuropathy

## Abstract

**Objectives:**

Sjögren's syndrome (SS) is a chronic inflammatory disease with an autoimmune background with possible complications from peripheral (PNS) and central nervous system (CNS). The aim of this study was to assess the prevalence and to describe the phenotype of peripheral neuropathies in patients with SS.

**Materials & Methods:**

We studied fifty patients with primary Sjögren's syndrome for peripheral nervous system involvement. All patients underwent neurological and rheumatological examination followed by nerve conduction studies (NCS) of nine peripheral nerves.

**Results:**

Thirty‐six patients (72%) fulfilled the criteria for the diagnosis of neuropathy. Carpal tunnel syndrome (54%) and axonal sensorimotor neuropathy (22%) were the most common. Neurological symptoms preceded the diagnosis of SS in eight patients.

**Conclusions:**

Peripheral neuropathies are frequent in SS patients. Neurologists should be aware of possible autoimmune causes of neuropathies because clinical manifestations of neuropathy may precede the development of other symptoms of the autoimmune disease.

## Graphical abstract

Our study shows that peripheral neuropathies are frequent among patients with primary Sjögren's syndrome. Neurologists should be aware of possible autoimmune causes of neuropathies because clinical manifestations of neuropathy may precede the development of other symptoms of the autoimmune disease.

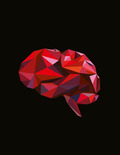

## 1 INTRODUCTION

Sjögren's syndrome (SS) is a chronic inflammatory disease with the autoimmune background. It is characterized by the presence of lymphocytic infiltrates in the exocrine glands (mainly salivary and lacrimal), which causes dryness of the mucosal surfaces (eyes, mouth, nose, throat, and vagina). Sjögren's syndrome may be primary (pSS)—it occurs then as an independent disease or secondary (sSS)—when it is associated with other systemic connective tissue diseases, most commonly rheumatoid arthritis. The prevalence of the pSS is estimated to be 0.2%–1% of the population (Binard et al., 2007; Westhoff & Zink, 2010). Despite the sicca symptoms, there are various extraglandular, organ‐specific manifestations of pSS, which play a major role in the prognosis.

Patients with pSS may present a wide spectrum of neurological manifestations, including both the peripheral (PNS) and central nervous system (CNS). CNS involvement has been reported in 6%–48% of pSS patients and PNS in 2%–60% of patients (Andonopoulos, Lagos, Drosos, & Moutsopoulos, 1990; Govoni et al., 1999; Hietaharju, Yli‐Kerttula, Häkkinen, & Frey, 1990; Lafitte et al., 2001; Mauch et al., 1994).

Peripheral neuropathy may be the first symptom, but may also develop later in the course of the disease. The following subtypes of peripheral polyneuropathy in SS were identified: three types of sensory neuropathies (sensory axonal, small‐fiber, and sensory ataxic), axonal sensorimotor polyneuropathy, mononeuropathy, multiple mononeuropathy, demyelinating polyradiculoneuropathy, cranial neuropathy, and autonomic neuropathy. The clinical course of the subtypes of peripheral neuropathy vary widely, but all of them may lead to significant pain and deterioration of quality of life in patients with pSS (Padua et al., 2005; Sopacua et al., 2019). The aim of this study was to assess the prevalence and to describe the phenotype of peripheral nervous system involvement in patients with pSS in tertiary care university hospital in Poland. To our knowledge, this is the first study in this subject in Polish population.

## 2 MATERIALS & METHODS

### 2.1 Patients

We studied a group of fifty consecutive patients with pSS from the Department of Internal Diseases, Connective Tissue Diseases and Geriatrics of the Medical University of Gdansk, Poland. Diagnosis of pSS was made upon criteria of American European Consensus Group from 2002 and criteria of Sjögren's International Collaborative Clinical Alliance from 2012 (Shiboski et al., 2012; Vitali, 2003). Patients with additional connective tissue diseases (secondary SS), as well as patients with diabetes, were excluded. All patients were evaluated by rheumatologist, and all clinical data were collected during medical history.

### 2.2 Neurological assessment and nerve conduction studies

All patients underwent clinical neurological examination including conventional investigation of sensory and motor functions, tendon reflexes, cranial nerve investigation, and nerve conduction studies (NCS). Neurological examination and NCS were performed and evaluated by one certified neurologist.

The NCS examinations were conducted following our standard laboratory methods in accordance with the recommended protocol of the American Association of Electrodiagnostic Medicine (AAEM) using Medtronic's Dantec Keypoint G4. Recordings were performed with skin temperature control 32–34°C. NCS was performed on nine peripheral nerves in each patient—peroneal, tibial, sural, median—bilaterally, and ulnar—unilaterally.

In motor NCS, we analyzed the following parameters: conduction velocity, distal latency, M‐wave amplitude, F‐wave latency, and frequency. In sensory NCS, conduction velocity and amplitude of sensory potentials were analyzed. The lower limits of normal values [sensory nerve and compound muscle action potential (SNAP and CMAP) amplitudes measured peak to peak] were determined from 50 age‐matched healthy individuals.

For the classification of polyneuropathies into axonal, demyelinating, or mixed, we used the ESTEEM (European Standardized Telematic tool to Evaluate Electrodiagnostic Methods) guidelines (Tankisi et al., 2005).

### 2.3 Laboratory data

Routine laboratory tests and following immunological parameters were recorded in all patients: serum protein electrophoresis; monoclonal gammopathy detected by immunofixation; C3 and C4 complement detected by nephelometry; serum cryoglobulins, measured after centrifugation and storage at 4°C for at least 72 hr; rheumatoid factor (RF) measured by nephelometry; ANA tested by IF using HEp‐2 cell substrate; antibodies to anti‐Ro/SS‐A and anti‐La/SS‐B assayed by ELISA method or detected by Western blot; anti‐Ro‐52 (detected by Western blot); erythrocyte sedimentation rate (ESR); c‐reactive protein (CRP); β‐2‐microglobulin; vitamin B12; fibrinogen; lactate dehydrogenase (LDH); creatine kinase (CK); and peripheral blood counts. Normal ranges for local laboratory were taken into account.

### 2.4 Disease activity and functional outcome

SS activity was assessed using the EULAR Sjögren's Syndrome Disease Activity Index (ESSDAI) (Seror et al., 2010). The ESSDAI consists of 12 domains: constitutional, lymphadenopathic, glandular, articular, cutaneous, pulmonary, renal, muscular, peripheral nervous system (PNS), central nervous system (CNS), hematological, and biological.

Organ complications were mainly diagnosed based on the ESSDAI.

For the needs of this study, some organ or system involvement, not included in ESSDAI, was defined as follows. Gastrointestinal tract involvement was defined as chronic atrophic gastritis, autoimmune hepatitis, or malabsorption syndrome after exclusion of accompanying celiac disease. Cardiovascular system involvement was defined as pericarditis or pulmonary arterial hypertension. CNS involvement was confirmed in imaging studies (computed tomography, magnetic resonance imaging, single‐photon emission computed tomography) or cerebrospinal fluid analysis.

We used EULAR Sjögren's Syndrome Patient Reported Index (ESSPRI) to assess the severity of patients' symptoms such as dryness, fatigue, and pain (Seror et al., 2011). To measure disease‐related damage, we used **Sjögren's** Syndrome Disease Damage Index (SSDDI) (Claudio Vitali, 2008).

For the assessment of clinical disability due to peripheral neuropathy, the Overall Disability Sum Score (ODSS) scale was used (Merkies et al., 2002). The ODSS scale consists of a checklist for interviewing the patient and focuses on limb functions. The score ranges from 0 to 5 in the upper limb and from 0 to 7 in the lower limb. A score of 0 indicates no limitations, and highest scores (5 or 7 depending on section) indicate inability to perform a purposeful movement.

The Ethics Committee of the Medical University of Gdansk approved the study, and all patients gave their written informed consent to participate in the study.

### 2.5 Statistical analysis

Statistical operations were performed with Statistica version 13 (TIBCO Software Inc.). Continuous variables were compared using nonparametric Mann–Whitney *U* test after assessing for normality with the Shapiro–Wilk test. Fisher's exact test was used to compare the categorical variables. Statistical significance was set at a *p* value of < .05.

## 3 RESULTS

Fifty subjects were included in the study, of which 48 were females aged 33 to 69 (average 53.6 ± 10.5 years) and two were males aged 42 and 67. The mean age at diagnosis of SS was 50.4 ± 14 years. 40 patients (80%) reported subjective symptoms indicative of peripheral nervous system involvement such as paresthesia or neuropathic pain. 36 patients (72%) met the criteria for the diagnosis of neuropathy. The distribution of subtypes of neuropathy is presented in Table 1.

**Table 1:** Peripheral nervous system involvement in pSS

Type of neuropathy	Number of patients (%)
Carpal tunnel syndrome	27 (54)
Axonal sensorimotor	11 (22)
Mononeuropathy	6 (12)
Cranial	4 (8)
Axonal sensory	1 (2)
Axonal motor	1 (2)
Small‐fiber neuropathy	1 (2)

Abbreviations: pSS, primary Sjögren's syndrome.

For the needs of the present study, we included 23 of 50 (46%) patients with neuropathies other than carpal tunnel syndrome (CTS) or patients with additional CTS into the group with peripheral nervous system involvement (further referred to as PNS+).

### 3.1 13 of 50 (26%) patients had only CTS, and 14 of 23 (61%) PNS+ patients had additional CTS

A pure small‐fiber neuropathy was suspected in three patients, but we did not have the possibility to conduct additional neurophysiological testing. All of them reported neuropathic pain present in lower and upper extremities. Two rated the pain at the level of 6 points on the VAS and one at the level of 7.

In one patient with normal NCS, the diagnosis of SFN was established based on abnormal clinical and quantitative sensory testing (QST) performed in another neurological laboratory.

In 8 (35%) of 23 PNS+ patients, neurological symptoms preceded the diagnosis of pSS.

The most common clinical presentations were paresthesias and neuropathic pain beginning in distal parts of extremities, sensory deficits in a “glove‐stocking” distribution, and mild‐to‐moderate muscle weakness reported by patients. The onset was usually chronic or subacute, and the course was predominantly slowly progressive.

The neurological examination revealed sensory deficits (superficial or deep), as well as diminished or absent tendon reflexes in the affected limbs. Muscle weakness was usually mild with a distal‐to‐proximal pattern.

In one patient, ataxic sensory neuropathy led to severe loss of proprioception and kinesthesia resulting in significant disability.

CNS involvement was confirmed in 12 patients (24%), and the following clinical presentations were observed: multiple sclerosis‐like syndrome, cerebral vasculitis, myelitis, meningoencephalitis, and cognitive impairment with abnormalities confirmed in the SPECT examination.

### 3.2 Comparison of clinical and laboratory data between patients with and without peripheral nervous system involvement in pSS

Clinical data were compared between patients with peripheral nervous system involvement (PNS+, *n* = 23) and the remaining 27 patients (PNS−). Table 2 summarizes the comparison of the groups, and Table 3 presents the laboratory data.

**Table 2:** Clinical comparison of PNS+ and PNS− pSS patients

Characteristics	PNS+, *n* = 23	PNS−, *n* = 27	*p*‐Value
Age (years, mean ± *SD*)	56.96 ± 11.38	54.81 ± 9.58	.323
First symptoms (year of life, mean ± *SD*)	45.83 ± 12.56	44.67 ± 11.17	.602
Diagnosis of pSS(year of life, mean ± *SD*)	53.52 ± 12.11	51.48 ± 11.01	.405
Time to diagnosis (years, mean ± *SD*)	7.7 ± 5.16	6.81 ± 7.2	.229
Disease duration (years, mean ± *SD*)	3.65 ± 3.42	3.78 ± 4.03	.938
Xerophthalmia	22 (96%)	27 (100%)	.273
Xerostomia	22 (96%)	27 (100%)	.273
Parotid enlargement	17 (74%)	12 (44%)	**.035**
Positive Schirmer test	17 (74%)	13 (48%)	.061
CNS involvement	7 (30%)	5 (19%)	.325
Vasculitis	7 (30%)	4 (15%)	.183
Articular involvement	12 (52%)	16 (59%)	.614
Gastrointestinal tract involvement	12 (52%)	14 (52%)	.981
Cardiovascular system involvement	2 (9%)	3 (11%)	.776
Respiratory tract involvement	15 (65%)	10 (37%)	**.047**
Lymphadenopathy	14 (61%)	5 (19%)	**.002**
Lymphoma	2 (9%)	0 (0%)	.073
Urinary tract involvement	12 (52%)	13 (48%)	.776
Autoimmune thyroid disease	2 (9%)	4 (15%)	.502
CNS involvement	7 (30%)	4 (15%)	.183
Cyclophosphamide treatment	4 (17%)	0 (0%)	**.010**
Steroid treatment	19 (83%)	18 (67%)	.194
ESSDAI (mean ± *SD*)	7 ± 6.82	4.7 ± 4.58	.245
ESSPRI (mean ± *SD*)	5.47 ± 1.66	4.82 ± 1.72	.187
SSDDI (mean ± *SD*)	4.65 ± 2.5	3.04 ± 2.1	.055
ODSS (mean ± *SD*)	1.78 ± 2.21	0.59 ± 0.93	.083

Abbreviations: CNS, central nervous system; ESSDAI EULAR, Sjögren's Syndrome Disease Activity Index; ESSPRI EULAR, Sjögren's Syndrome Patient Reported Index; ODSS, Overall Disability Sum Score; PNS−, patients without peripheral neuropathy; PNS+, patients with peripheral neuropathy; pSS, primary Sjögren's syndrome; SSDDI, Sjögren's Syndrome Disease Damage Index.

The results with statistical significance *p*<.05 are shown in bold.

**Table 3:** Laboratory data comparison of PNS+ and PNS− pSS patients

Characteristics	PNS+ *n*=23	PNS− *n* = 27	*p*‐Value
Positive ANA	23 (100%)	27 (100%)	‐
Positive anti‐Ro/SS‐A	16 (70%)	19 (70%)	.979
Positive anti‐La/SS‐B	10 (43%)	13 (48%)	.648
Rheumatoid factor	13 (57%)	18 (67%)	.665
Cryoglobulins	3 (13%)	7 (26%)	.668
Hypocomplementemia	6 (26%)	2 (7%)	**.045**
Hypergammaglobulinemia	13 (57%)	15 (56%)	.429
Hypogammaglobulinemia	1 (4%)	4 (15%)	.229
Monoclonal gammopathy	1 (4%)	1 (4%)	.543
Anemia (Hb < 12g/dl)	9 (39%)	10 (37%)	.879
Leukopenia (WBC < 4,000/mm^3^)	7 (30%)	11 (41%)	.449
Thrombocytopenia (PLT < 150,000/mm^3^)	3 (13%)	2 (7%)	.508
Hyperfibrinogenemia	14 (61%)	12 (44%)	.246
B−2‐microglobulin > 2.6 mg/L	12 (52%)	11 (41%)	.418
Positive anti‐CCP	0 (0%)	4 (41%)	.054
ESR > 30 mm/hr	6 (26%)	12 (44%)	.178
CRP > 5 mg/L	4 (17%)	6 (22%)	.670

Abbreviations: ANA antinuclear antibodies; CRP, C‐reactive protein; ESR, erythrocyte sedimentation rate; PNS−, patients without peripheral neuropathy; PNS+, patients with peripheral neuropathy; pSS, primary Sjögren's syndrome.

The results with statistical significance *p*<.05 are shown in bold.

The mean time between the onset of sicca symptoms and the diagnosis of pSS was 7,7 ± 5,16 years and 6,81 ± 7,2 years in the PNS+ and PNS− groups, respectively (*p* = .229).

We observed more often salivary gland enlargement (74% vs. 44% *p* < .05), lymphadenopathy (61% vs. 19% *p* < .05), and respiratory tract involvement (65% vs. 37% *p* < .05) in the PNS+ group. Those patients were also more often treated with cyclophosphamide (*p* < .05).

Considering the laboratory results, the groups differed only in the incidence of hypocomplementemia (26% vs. 7% *p* < .05). There was no statistically significant difference between the two groups in involvement of other organs, laboratory data, or disease duration.

We also studied the differences between individual subtypes of neuropathy. We found that patients with sensorimotor neuropathy had longer disease duration (5,82 ± 3,82 versus 1,67 ± 1,07 *p* < .05), more of them had anti‐SS‐A antibodies (91% vs. 50% *p* < .05), hypocomplementemia (55% vs. 0% *p* < .05), hypergammaglobulinemia (82% vs. 33% *p* < .05), and elevated levels of B‐2‐microglobulin (91% vs. 17% *p* < .05). The comparison is presented in Table 4.

**Table 4:** Laboratory data comparison of pSS patients with sensorimotor neuropathy and other neuropathies

Characteristics	Sensorimotor neuropathy (*n* = 11)	Other neuropathies (*n* = 12)	*p*‐Value
Disease duration (years, mean ± *SD*)	5.82 ± 3.82	1.67 ± 1.07	**.012**
Positive anti‐Ro/SS‐A	10 (91%)	6 (50%)	**.044**
Positive anti‐La/SS‐B	7 (63%)	3 (25%)	.073
Cryoglobulins	3 (27%)	0 (0%)	.093
Hypocomplementemia	6 (55%)	0 (0%)	**.004**
Hypergammaglobulinemia	9 (82%)	4 (33%)	**.025**
B−2‐microglobulin > 2.6 mg/L	10 (91%)	2 (17%)	**.00055**
ESSDAI (mean ± *SD*)	7.18 ± 7.1	6.83 ± 6.87	.927
SSDDI (mean ± *SD*)	5.73 ± 3	3.67 ± 1.44	.133
ODSS (mean ± *SD*)	2.36 ± 1.8	1.25 ± 2.49	.051

Abbreviations: ESSDAI EULAR, Sjögren's Syndrome Disease Activity Index; ODSS, Overall Disability Sum Score; pSS, primary Sjögren's syndrome; SSDDI, **Sjögren's** Syndrome Disease Damage Index.

The results with statistical significance *p*<.05 are shown in bold.

Although lymphadenopathy was comparable between the subgroups, of the two patients with lymphoma (4% of our group), both had sensorimotor neuropathy.

## 4 DISCUSSION

Primary Sjögren's syndrome remains under‐recognized despite being a highly prevalent autoimmune disease. The clinical presentation of this disorder extends from dryness to systemic involvement, including peripheral nervous system. Despite the progress of knowledge about the SS, there is a large discrepancy between authors in the assessment of the prevalence and symptomatology of the nervous system involvement in this group of patients. Pathogenesis is not fully understood, and there are no uniform recommendations regarding treatment. Awareness of the wide range of neurological symptoms accompanying SS is important due to impact on the deterioration of quality of life, potential complications, but also the possibility of significant improvement after immunosuppressive treatment.

Our study shows a high prevalence (46%) of peripheral nervous system involvement in a cohort of patients with pSS in a single hospital. Among polyneuropathies, sensorimotor neuropathies were the most common. SS develops in 0.2 to 1% of the population, and it seems to be as frequent as rheumatoid arthritis. In previous studies, the peripheral nervous system involvement was observed in 2%–60% of SS patients and sensory neuropathies were most common (Andonopoulos et al., 1990; Delalande et al., 2004; Grant, Hunder, Homburger, & Dyck, 1997; Mori et al., 2005).

It is worth noting that 80% of our subjects reported subjective symptoms suggestive of polyneuropathy, while final diagnosis was made only in 46%. That means that in clinical settings, diagnosis of SS‐related polyneuropathy should not be based solely upon history or questionnaires.

Some of the previous studies suggest that nervous system involvement can be the presenting feature of SS, while others report that it is rather a late finding in the course of the disease (Govoni et al., 1999; Grant et al., 1997; Griffin et al., 1990; Malinow et al., 1986; Mori et al., 2005; Vrethem et al., 1990). Berkowitz et al. found that neurological symptoms preceded the diagnosis of SS in 81% of patients (Berkowitz & Samuels, 2014). In another study, conducted by Seeliger et al., a diagnosis of Sjögren's syndrome was made in 44 of 184 (24%) patients with symptoms and signs of a severe progressive symmetric or asymmetric peripheral neuropathy (Seeliger et al., 2019). In our group, neurological symptoms preceded the diagnosis of pSS in 35% of patients. This fact implies that these are patients who will first report to a neurologist. In our region, such patients, in case of suspected or diagnosed systemic connective tissue disease, are referred from the neurological ward for further immunosuppressive treatment in the rheumatological department.

Compared to the PNS− group, the PNS+ group had significantly higher proportions of patients with salivary gland enlargement, respiratory tract involvement, lymphadenopathy, and hypocomplementemia, and patients treated with cyclophosphamide due to extraglandular manifestations.

Previous studies suggest an association between neurological involvement and vasculitis, lung involvement, and constitutional symptoms such as fever and fatigue (Binder, Snaith, & Isenberg, 1988; Gono et al., 2011; Miwa et al., 2003; Teixeira et al., 2013).

Unlike other authors (Jamilloux et al., 2014; Miwa et al., 2003), we did not find specific immunological markers of B‐cell activation or monoclonal proliferation between PNS+ and PNS− group.

Chronic B‐cell activation is characterized by elevated levels of serum gamma‐globulins (mainly IgG), presence of other B‐cell produced serologic markers (ANA, anti‐Ro/SS‐A, anti‐La/SS‐B antibodies, RF), and the formation of ectopic lymphoid tissue with germinal center‐like structures. Another immunological sign of pSS is the predisposition to developing oligo‐ and monoclonal B‐cell proliferation, characterized by the presence of mixed cryoglobulinemia, monoclonal gammopathy, and B‐cell lymphomas (Hansen, Lipsky, & Dörner, 2007).

In our study, only patients with sensorimotor neuropathy had elevated markers of high B‐cell activation as mentioned above. But they also had a significantly longer disease duration compared to other subgroups.

Sensorimotor neuropathies were so far linked to higher prevalence of chronic B‐cell activation markers and B‐cell monoclonal proliferation, while nonataxic sensory neuropathies were characterized by less frequent chronic B‐cell activation suggesting another causal mechanism (Chai, Herrmann, Stanton, Barbano, & Logigian, 2005; Mori et al., 2005; Ramos‐Casals et al., 2008; Sène et al., 2011).

Our hypothesis is that sensorimotor neuropathies are the next step in the development of neuropathy in pSS, preceded by pure sensory neuropathies, and therefore, they are associated with a higher incidence of chronic B‐cell activation corresponding to the duration of the disease.

Carpal tunnel syndrome (CTS) was a frequent finding in our study (54%). So far, this type of neuropathy has been rarely observed in pSS patients, although it is a common neuropathy in the general population and its frequency increases with age. Estimated prevalence of CTS in the general population is 1 to 5% with a higher incidence in women than in men (female‐to‐male ratio is approximately 3:1) (Atroshi et al., 1999; Burton, Chen, Chesterton, & van der Windt, 2018; Pourmemari, Heliövaara, Viikari‐Juntura, & Shiri, 2018).

Binder et al. (1988) mention that CTS was diagnosed in 6 of 105 patients included in their study, three of them had the SS secondary to the rheumatoid arthritis. In our study, the prevalence of CTS was associated with the subgroup of pSS patients with articular involvement. Our hypothesis is that those patients can develop CTS resulting from inflammation in the joint and overgrowth of the synovium. Clarification of the CTS etiology and course in pSS requires further research.

Extraglandular manifestations of Sjögren's syndrome seem to be underdiagnosed.

Frequent occurrence of neurological manifestations related to both the central and peripheral nervous system, and the fact that neurological complications may precede the occurrence of typical symptoms of dryness, makes this topic very essential for every neurologist. It is important to highlight that neurologist may be the first specialist to assess the patient and to determine how quickly the diagnosis of systemic diseases will be established. That is why the SS should be considered in differential diagnosis of autoimmune peripheral neuropathies.

### 4.1 Treatment

Currently, there are no guidelines based on randomized trials regarding the treatment of patients with SS with nervous system involvement. Patients with oligosymptomatic and self‐limiting course of the disease do not require pharmacological treatment. However, in case of high disease activity and the detected progression of lesions, immunosuppressive treatment is applied. The use of corticosteroids and intravenous cyclophosphamide pulses is most often recommended. Cyclosporine, azathioprine, and methotrexate also seem to be effective (Govoni, Padovan, Rizzo, & Trotta, 2001; Soliotis, Mavragani, & Moutsopoulos, 2004). Intravenous administration of immunoglobulins (IVIGs) in the induction or adjunctive treatment of peripheral neuropathy gives favorable results (Kizawa et al., 2006; Wakasugi et al., 2009). In the literature, there are also descriptions of effective treatment with infliximab (Mavragani & Moutsopoulos, 2007), rituximab (Gorson, Natarajan, Ropper, & Weinstein, 2007; Sève et al., 2007), and tacrolimus (Fukuda et al., 2009). In some patients, very good results can be obtained after plasmapheresis with simultaneous immunosuppressive treatment (Soliotis et al., 2004).

In the treatment of neuropathic pain, antiepileptic drugs, tricyclic antidepressants, and opioids are used (Hoitsma et al., 2004).

Our study has some limitations. Main limitation is the small number of patients with pSS. Secondly, this study was conducted in a tertiary care university hospital; thus, referral bias may have played a role in patient selection. Additionally, we did not have the possibility to conduct neurophysiological testing in cases where a pure small‐fiber neuropathy was suspected.

The advantage is that our study, unlike many others, is a prospective study with a thorough neurological and rheumatological assessment performed by one certified specialist. We also excluded patients with additional connective tissue diseases, as well as patients with diabetes, which may be a potential complication of steroid therapy.

In conclusion, involvement of the PNS in SS patients seems frequent but remains underestimated. Rheumatologists should be more aware of the risk of neurological complications in pSS patients. Neurologists should be aware of possible autoimmune causes of neuropathies because clinical manifestations of neuropathy may precede the development of other symptoms of the autoimmune disease.

NCS is a noninvasive procedure, which should be used in diagnostics and follow‐up in this group of patients.

Guidelines for the diagnostics and treatment of peripheral neuropathies in patients with pSS are needed.

## CONFLICT OF INTEREST

None of the authors has any conflict of interest to disclose.

## AUTHOR CONTRIBUTION

Marta Jaskólska, Magdalena Chylińska, Anna Masiak, Mariusz Siemiński, Zenobia Czuszyńska, Żaneta Smoleńska, and Zbigniew Zdrojewski conceptualized the study. Marta Jaskólska, Magdalena Chylińska, Anna Masiak, Żaneta Smoleńska, Mariusz Siemiński, Marcin Ziętkiewicz, Zenobia Czuszyńska, and Zbigniew Zdrojewski contributed to methodology. Marta Jaskólska, Magdalena Chylińska, Mariusz Siemiński, and Marcin Ziętkiewicz performed formal analysis and investigation. Marta Jaskólska, Anna Masiak, Mariusz Siemiński, Magdalena Chylińska, and Marcin Ziętkiewicz wrote and prepared the original draft. Marta Jaskólska, Magdalena Chylińska, Anna Masiak, Żaneta Smoleńska, Mariusz Siemiński, Marcin Ziętkiewicz, Zenobia Czuszyńska, and Zbigniew Zdrojewski wrote, reviewed, and edited the manuscript. Zbigniew Zdrojewski underwent supervision.

## Data Availability

Data are available on request due to privacy/ethical restrictions.
